# Segmental ileal dilatation with supernumerary intestinal muscle coat in a neonate

**DOI:** 10.1186/s40792-015-0022-8

**Published:** 2015-02-17

**Authors:** Tutku Soyer, Beril Talim, Feridun Cahit Tanyel

**Affiliations:** Department of Pediatric Surgery, Faculty of Medicine, Hacettepe University, 06100 Ankara, Turkey; Department of Pediatrics, Pathology Unit, Faculty of Medicine, Hacettepe University, Ankara, Turkey

**Keywords:** Segmental ileal dilatation, Neonate, Gastrointestinal smooth muscle

## Abstract

Segmental intestinal dilatation (SID) is a rare developmental anomaly of the midgut, characterized by sharply demarcated dilatation of a gastrointestinal segment with clinical findings of intestinal obstruction. Although morphologic criteria for SID are clearly delineated, etiological origin of dilated intestine is unknown. Histology of the resected segment is usually reported to have normal presence of ganglion cells in the myenteric and submucosal plexuses. Intestinal muscle is hypertrophied, and heterotopic gastric mucosa may also be encountered. A 3-day-old male infant presenting with clinical findings of intestinal obstruction was diagnosed to have SID and had supernumerary intestinal muscle coat (SIMC) in the dilated segment, without any evidence of neurological abnormality. Histopathological findings of the resected ileal segment are reported to discuss the role of architectural malformation of muscularis propria as a cause of SID.

## Background

Segmental intestinal dilatation (SID) is a rare gastrointestinal anomaly introduced by Rossi and Giocomoni [1]. According to the criteria proposed by Swenson and Rathauser, SID is defined as limited bowel dilatation with three- to fourfold increased size, with an abrupt transition between dilated and normal bowel, without any intrinsic and extrinsic barrier distal to the dilatation or abnormal neurological innervation [2]. Approximately half of the SID cases are seen in the first days of life, and clinical findings of intestinal obstruction are the most common presentation [3]. Anemia, failure to thrive, and abdominal pain are usually encountered in older children [3].

Although diagnostic criteria of SID are well established, the etiology of the disease is unclear. Histology of the involved bowel usually shows normal neurological innervation with a hypertrophied or thin muscle layer [4]. Heterotopic esophageal and gastric mucosa or cartilaginous foci are also reported. However, none of the histopathological findings are able to explain the etiology of SID.

The presence of an additional smooth muscle layer in the intestine is a rare congenital anomaly of enteric smooth muscle and considered as a cause of pseudo-obstruction or constipation [5]. Supernumerary intestinal muscle coat (SIMC) has been reported in patients with Hirschsprung disease and intestinal pseudo-obstruction. However, it has not been identified in the resected bowel specimens of patients with SID previously.

A 3-day-old male infant with intestinal obstruction was diagnosed as SID, and the patient underwent resection of involved bowel and anastomosis. Microscopical evaluation of the resected segment revealed SIMC with normal neurological innervation. We aim to discuss the role of architectural malformation of intestinal muscle coat in the etiology of SID.

## Case presentation

A 20-day-old male infant, weighing 3,200 g, was delivered vaginally at 37 weeks of gestation. He was admitted to the emergency department with abdominal distention and bilious vomiting. He had inability to pass stool in the first 3 days of his life. Then, he irregularly passed the stools with rectal stimulation. His prenatal history was unremarkable. He has no family history of pseudo-intestinal obstruction.

**Figure 1:**
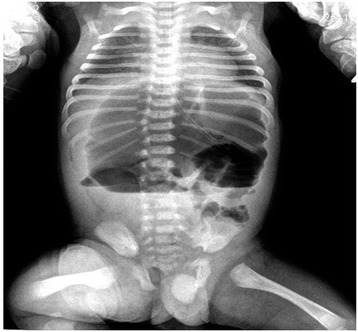
**Abdominal X-ray showing a distended large bowel loop with air-fluid levels.**

**Figure 2:**
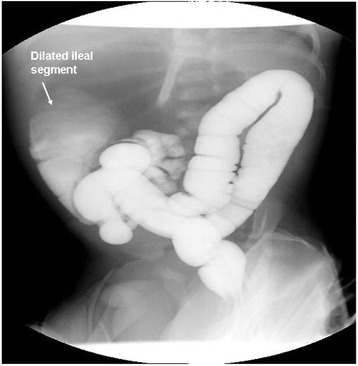
**Contrast enema showing segmental dilatation of the ileum with normal colon.**

**Figure 3:**
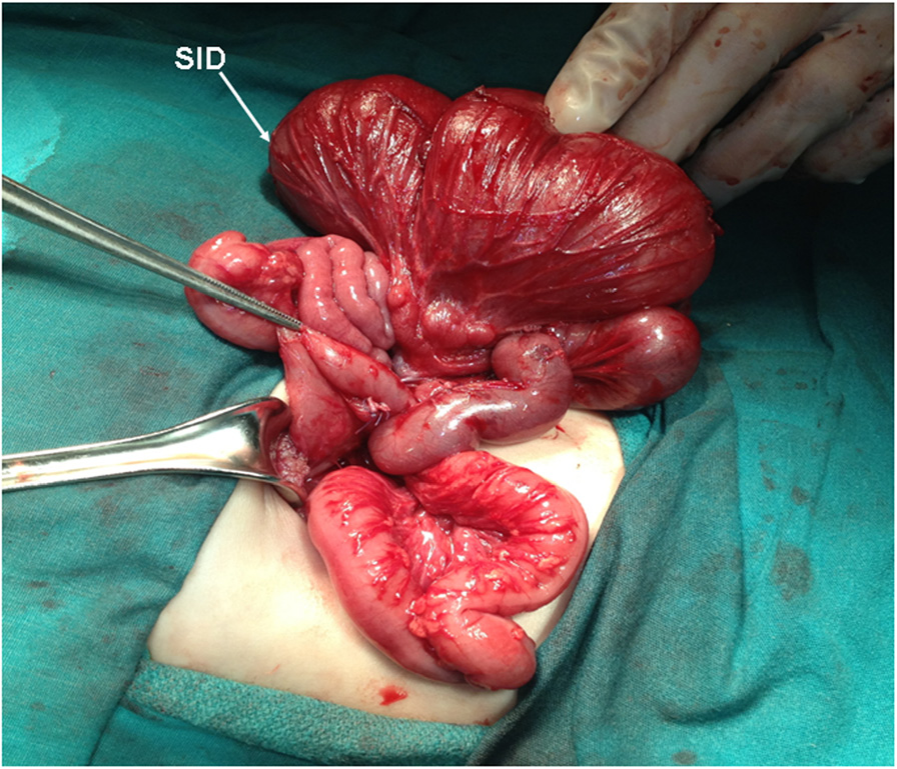
**Fivefold dilated ileal segment with malrotation.**

On admission, his height and weight were at 50th percentile, axillary temperature was 37°C, pulse was 130/bpm, and blood pressure was 60/45 mmHg. Physical examination was normal except a distended abdomen. Laboratory findings revealed hemoglobin 13.5 g/dL, hematocrit 33.2%, leukocyte 10.2 × 10^3^/μL, and platelet 310 × 10^3^/μL. Biochemical analyses including liver and renal function tests were within normal limits. The physical examination of all systems was normal except abdominal distention. His abdominal X-ray showed a distended large bowel loop with air-fluid levels (Figure 1). He underwent colon contrast enema with presumptive diagnosis of Hirschsprung disease and was diagnosed as segmental dilatation of ileum with normal colon (Figure 2). In surgical exploration, a dilated ileal segment of 15 cm was detected 25 cm distal to the duodenum and 30 cm proximal to the terminal ileum. This segment was fivefold dilated, and neither extrinsic nor intrinsic barrier was found as a cause of the dilatation (Figure 3). The dilated segment of the ileum was resected, and the bowel continuity was achieved by end-to-end anastomosis. During exploration, the cecum was found at the left upper quadrant, and malrotation of intestines was detected. Ladd bands between the duodenum and colon were excised, and Ladd procedure was completed with appendectomy.

**Figure 4:**
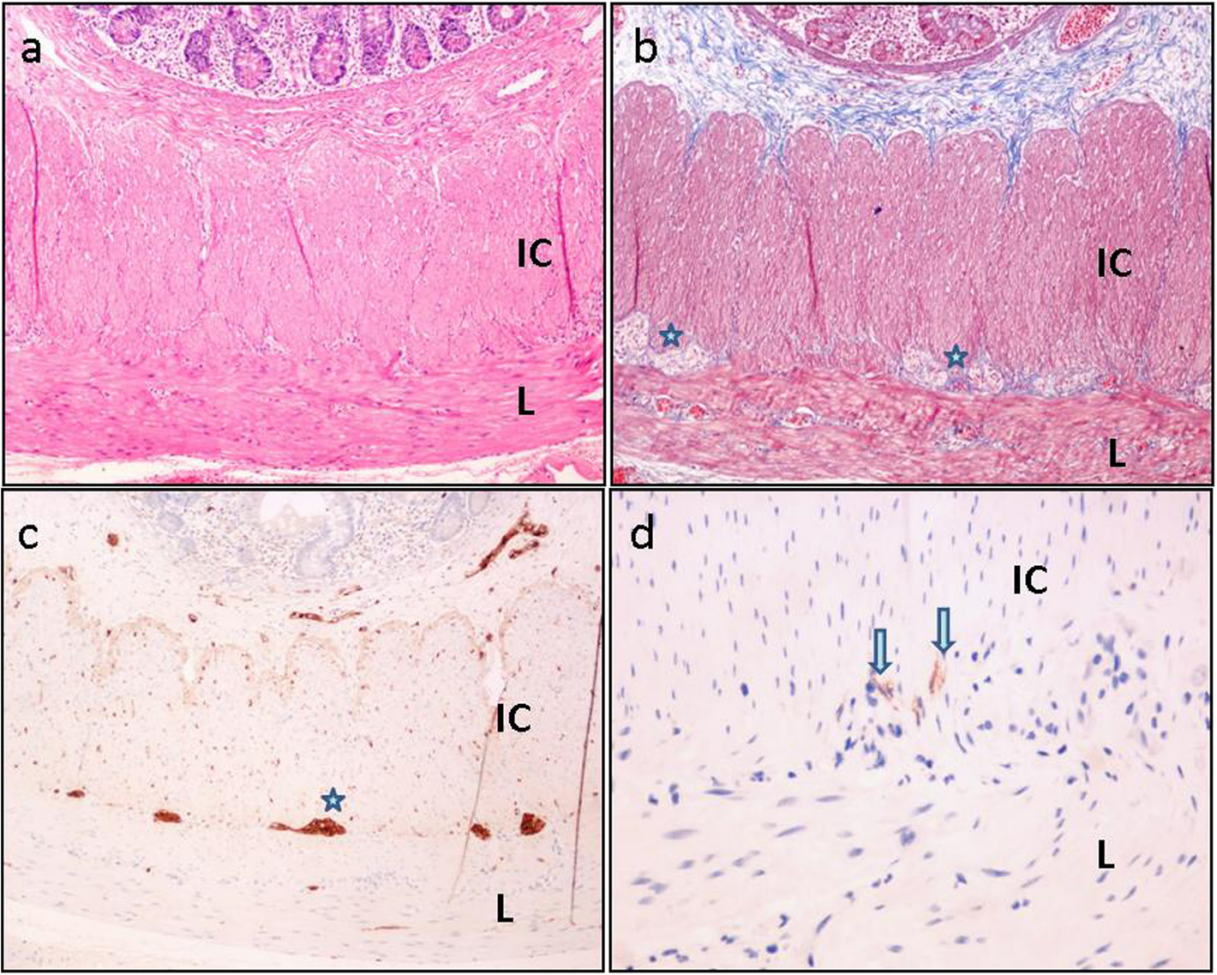
**Microscopic evaluation of the non-dilated intestinal segment.** Sections from the non-dilated segment showing normal architecture consisting of inner circular (IC) and outer longitudinal (L) smooth muscle layers. Ganglia and nerves (*stars*) as well as interstitial cells of Cajal (*arrows*) are non-remarkable. **(a)** Hematoxylin and eosin (H&E), **(b)** Masson trichrome, **(c)** S-100, and **(d)** CD117 (c-kit). **(a-c)** Original magnification × 10. **(d)** Original magnification × 40.

**Figure 5:**
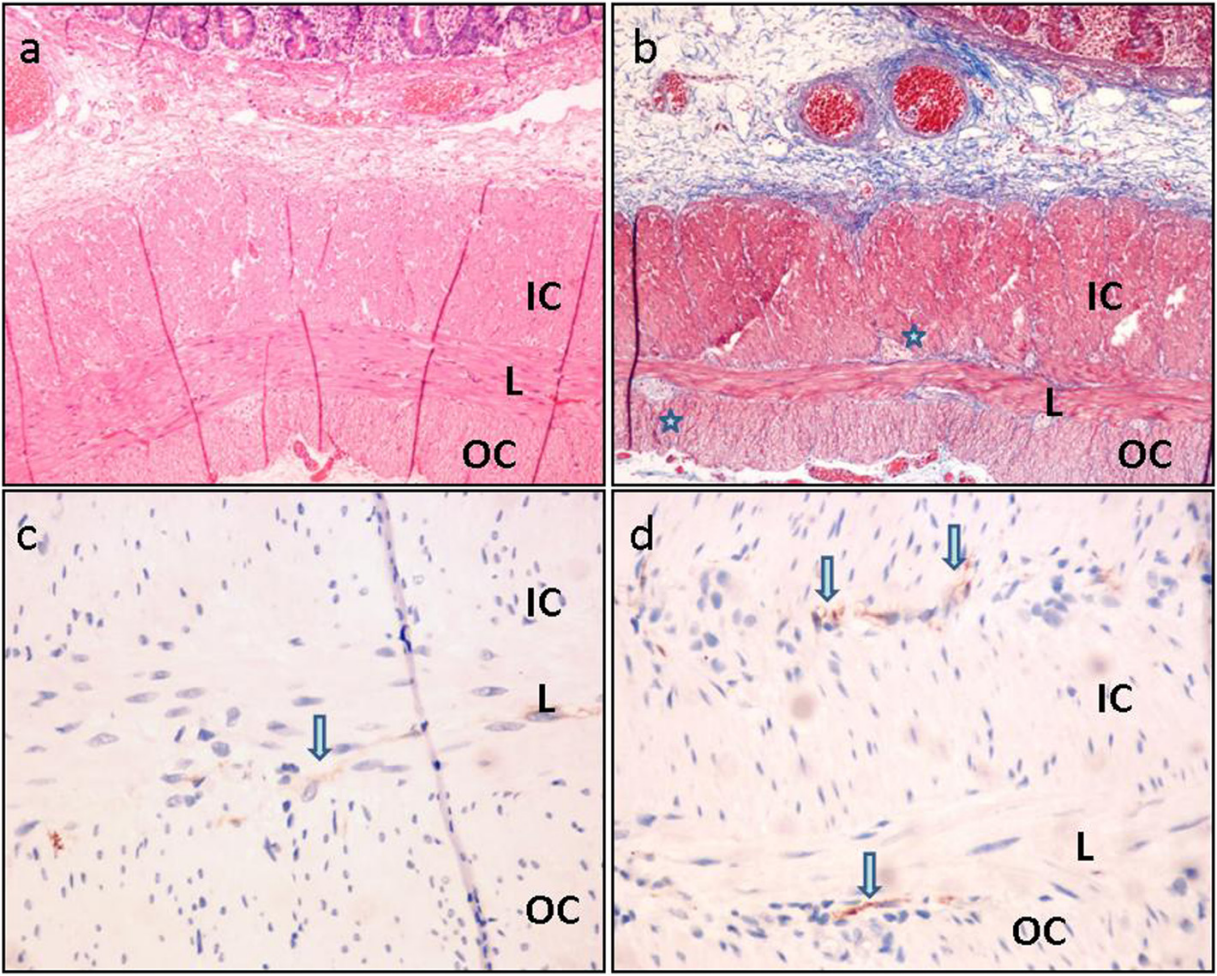
**Sections from the dilated segment.** In addition to the inner circular (IC) and longitudinal (L) layers, there is an extra outer smooth muscle layer (OC). Ganglia and nerves (*stars*) **(b)** and interstitial cells of Cajal (*arrows*) **(c, d)** are located on both sides of the longitudinal muscle. **(a)** Hematoxylin and eosin (H&E), **(b)** Masson trichrome, and **(c-d)** CD117 (c-kit). **(a-b)** Original magnification × 10. **(c-d)** Original magnification × 40.

Macroscopic examination of the 15-cm resection specimen revealed a dilated segment of 10 cm in length and 4 cm in diameter with sudden transition to normal intestinal diameter on both sides. Mucosal surface was intact. Microscopic evaluation of the non-dilated intestinal segment showed normal histology with inner circular and outer longitudinal smooth muscle layers of muscularis propria with normally distributed myenteric neural elements and ganglion cells and interstitial cells of Cajal, as shown by c-kit (CD117) immunohistochemical staining (Figure 4). In the sections from the dilated segment, in addition to the unremarkable inner circular smooth muscle and the adjacent longitudinal layer, an extra smooth muscle layer was present in the outermost side of muscularis propria, next to serosa (Figure 5). This additional external smooth muscle coat showed irregular thickness and haphazard orientation, mostly longitudinally arranged but showing oblique fascicles in some areas. Myenteric plexus and ganglion cells were normally placed between the inner circular and longitudinal muscle layers but were also present between the longitudinal and the outermost extracircular smooth muscle layers (Figure 5b). Interstitial cells of Cajal were detected on both sides of the longitudinal muscle (Figure 5c,d). There was no vacuolar change in the smooth muscle layer. Mucosa was unremarkable. The appendix was normal histologically. He was fed orally at the fifth postoperative day and discharged from the hospital after an uneventful period of 8 days. He has been followed up for the last 8 months, and he is free of symptoms.

## Discussion

SID is a rare congenital anomaly characterized by sharply demarcated dilatation of gastrointestinal tract without any evidence of mechanical obstruction [3]. The most common localization of dilated segment is the ileum and followed by the colon, jejunum, and duodenum [6,7]. Approximately half of the SIDs present in the first day of life [8]. Rarely, some cases become symptomatic during infancy and adolescence. SID is encountered in both sexes with a slight male predominance [3]. The most common clinical presentation is intestinal obstruction, and abdominal pain, anemia, and failure to thrive can be seen in older children [3]. Anemia is the first sign of SID in patients with heterotopic gastric mucosa. It has been reported that one third of SID cases have been detected incidentally during laparotomy [8]. Omphalomesenteric remnants and malrotation are reported as associated anomalies in SID. Malrotation associated with SID was also recognized in our patient during laparotomy.

Although diagnostic criteria are well established, the etiology of the disease is unclear. It has been suggested that transient embryonic structures such as vitelline vessels and omphalomesenteric bands cause extrinsic compression of both ends of bowel [9,10]. In addition, *in utero* vascular accidents, primary aplasia of an intestinal segment, and congenital damage to the nerves in myenteric plexus are considered as possible causes of SID [11]. The role of enteric nervous system and interstitial cells of Cajal was also investigated, and no neurogenic etiology could be attributed to SID [12]. We have also detected normal distribution of interstitial cells of Cajal in our case.

SID can also be detected as an intra-abdominal cystic mass in prenatal ultrasonography [13]. On abdominal X-ray, it is associated with dilated bowel loops with or without air-fluid levels and can be easily misdiagnosed as other causes of intestinal obstruction [3]. As in our case, contrast enema studies can be performed to differentiate other gastrointestinal anomalies. Contrast enema studies show marked segmental dilatation of small bowel loop with normal colon findings. Since most of the neonates undergo contrast enema studies, the use of computed tomography in the diagnosis of SID is limited and reserved for older patients.

The treatment of SID is resection of dilated segment and end-to-end anastomosis [3]. Most patients have uneventful course, and prognosis is excellent after surgical resection.

In most of the cases, histology of the resected segment is usually normal [3]. However, some of the cases showed hypertrophied or very thin muscle layer in the involved segment in histopathological evaluation. Heterotopic esophageal or gastric mucosa or cartilaginous foci are also reported in dilated intestinal segment [3]. Kobayashi et al. suggested that ectopic gastric mucosa causes interruption in the neural and muscular network in the dilated intestine [14]. However, all resected segments show normal presence of ganglion cells in myenteric and submucosal plexuses [6]. Histopathological recognition of localized vacuolization of the smooth muscle layer has suggested that myopathy might contribute to the pathogenesis of SID [6]. However, there are no reports of additional smooth muscle layer in the involved segment of SID in the literature. In the case reported here, no mucosal abnormality was present, ganglia and plexuses were normal in distribution, and there was no vacuolization in smooth muscle layer.

Architectural malformations of muscularis propria consist of supernumerary and/or haphazard orientation of muscle fibers with an additional neural plexus. Smith et al. have described supernumerary muscle coat in five patients and classified them as diffuse or segmental [15]. SIMC is also reported in a patient with Hirschsprung disease and considered as a congenital anomaly rather than an acquired histopathological alteration [5]. X-linked intestinal pseudo-obstruction due to filamin A mutation should be also considered in the differential diagnosis of SMIC. Kapur et al. reported FLNA mutations with intestinal pseudo-obstruction. They usually show male predominance, short small intestinal length, and malrotation [16]. FLNA mutations include abnormal intestinal layering throughout a longer segment of the intestine with neurologic impairment in histology. In our patient, abnormal layering is limited to a short segment of the intestine, with normal ganglia and without associated neurologic disease. In our case with SID, we have found segmental SIMC in the dilated segment. There was no vacuolar change in the muscle layers, but haphazard orientation was remarkable in some areas. Myenteric plexuses and ganglion cells were normally placed between the inner circular layer and the longitudinal layer, but some plexuses were also present between the longitudinal layer and the outermost circular extra smooth muscle layer. Although the role of myopathy was suggested in the etiology of SID, SIMC as a cause of SID has not been reported previously. We suggest that hypertrophied muscle or very thin muscle layer should be considered as result rather than the cause of SID [10]. SIMC or architectural malformation of the intestinal smooth muscle should be considered as the most likely cause of segmental dilatation. Presence of myenteric plexuses and ganglion cells towards the external surface also supports that this is a primary developmental problem.

## Conclusions

SID is a congenital anomaly of gastrointestinal tract with clinical findings of intestinal obstruction. We show for the first time that at an extremely rare malformation of muscularis propria, SIMC can be seen in the dilated segment of SID, and we suggest that this additional muscle layer can play a role in the etiology of SID.

## Consent

Written informed consent was obtained from the patient for publication of this case report and any accompanying images. A copy of the written consent is available for review by the Editor-in-Chief of this journal.

## Abbreviations

SID: segmental ileal dilatation
SMIC: supernumerary intestinal muscle coat
